# *Mycobacterium tuberculosis* peptide E7/HLA-DRB1 tetramers with different HLA-DR alleles bound CD4^+^ T cells might share identical CDR3 region

**DOI:** 10.1038/s41598-018-28344-7

**Published:** 2018-07-02

**Authors:** Yichuan Gan, Cong Wang, Yimin Fang, Yanan Yao, Xiaoxin Tu, Jiao Wang, Xi Huang, Yaoju Tan, Tao Chen, Kouxing Zhang, Yanming Shen, Lin Zhou, Jianxiong Liu, Xiaomin Lai

**Affiliations:** 1 Department of Microbiology, Zhongshan School of Medicine, Sun Yat-sen University, 74 Zhongshan Road II, Guangzhou, 510080 China; 2 China Ministry of Education Key Laboratory of Tropical Diseases Control, Tuberculosis Research Institute, Zhongshan School of Medicine, Sun Yat-sen University, 74 Zhongshan Road II, Guangzhou, 510080 China; 3 Gangdong Provincial Department of Education Key Laboratory of Functional Molecules from Marine Microorganisms, Gangdong Provincial Research Center for Severe Infectious Disease Prevention and Control Technology, Zhongshan School of Medicine, Sun Yat-sen University, 74 Zhongshan Road II, Guangzhou, 510080 China; 4 State Key Laboratory of Respiratory Disease of China, Guangzhou Chest Hospital, 62 Hengzhigang Road, Guangzhou, 510095 China; 5 Department of Immunology, Zhongshan School of Medicine, Sun Yat-sen University, 74 Zhongshan Road II, Guangzhou, 510080 China; 6 Tuberculosis Control Center of Guangdong Province, 485 West Huangpu Avenue, Guangzhou, 510630 China; 7 Third Affiliated Hospital of Sun Yat-sen University, 600 Tianhe Road, Guangzhou, 510630 China

## Abstract

Human CD4^+^ T cells play an important role in the immune response to *Mycobacterium tuberculosis* (MTB). However, little is known about the spectratyping characteristics of the CD4^+^ T-cell receptor (TCR) α- and β-chains CDR3 region in tuberculosis (TB) patients. We sorted MTB peptide E7-bound CD4^+^ T cells by using E7/HLA-DR tetramers constructed with different HLA-DRB1 alleles and extracted the CDR3 amino-acid sequences of TCR α- and β-chains. The results showed that the CDR3 sequences of E7-bound CD4^+^ T cells were completely or partially identical in a single patient. The sequences of MTB peptide C5-bound CD4^+^ T cells shared another, and non-peptide bound CD4^+^ T cells, as well as unbound CD4^+^ T cells with tetramers were different from each other. Specifically, diverse CDR3 sequences of E7-bound CD4^+^ T cells displayed similar protein tertiary structure in one TB patient. In summary, the TCR α- and β-chains of CDR3 lineage of CD4^+^ T cells in TB patients apparently drifted, and the predominant CDR3 sequences of TCR α- and β-chains that recognized the MTB antigen exhibited peptide specificity, and certain HLA-DR restriction was also established. This study elucidates the possible causes and mechanisms of peptide-specific CD4^+^ T-cell-related presentation against MTB.

## Introduction

Tuberculosis (TB) is one of the most widespread chronic infectious diseases mainly presented as a respiratory infection caused by *Mycobacterium tuberculosis* (MTB). While the mechanisms leading to loss of immune defense and disease reactivation are yet unknown, it is well established that CD4^+^ T cells are critical in controlling TB infection^1–5^. T cells recognize antigens by their T-cell receptors (TCRs), a process limited by the histocompatibility complex (MHC)^6^. CD4^+^ T cells are activated by recognizing the antigen peptide/MHC class II complex and induce a series of immunological reactions^7^. The human leukocyte antigen (HLA) gene is a set of complicated complex components and structures, the most important feature of which is its high polymorphism. The HLA-II class loci are in the HLA-D region, which includes HLA-DQ, DP, and DR sub-regions. The complex of HLA-DR (human leukocyte antigen-antigen D-related) and its ligand, a peptide composed of nine or more amino-acids, constitutes a ligand for the TCR. T cells expressing αβ receptors play an important role in immunization to MTB. High expansions of αβ T cells within the TCR repertoire also have been shown to occur in various diseases, such as cancers and immunological disorders, as well as in inflammatory and infectious diseases^8–10^. Furthermore, each Vα- (variable region of α-chain) or Vβ-chain constitutes three loops called the complementarity determining regions (CDRs) 1, 2, and 3, which interact with the peptide/MHC molecule. CDR1 and CDR2 regions recognize and bind to the side walls of an antigen in antigen-binding-groove of MHC molecules, whereas the CDR3 region is directly combined with antigenic peptides. Thus, the TCR specificity is mainly determined by its CDR3 region. In other words, analyzing and comparing the length and sequence of the CDR3 region can be judged as an indicator of T-cell clones^11^.

In 1996, Altman established a peptide/MHC tetramer assay using the principle of biotin-avidin cascade amplification, and greatly increased the intermolecular affinities and stability of MHC/peptide-TCR binding^12^. In our previous work, we showed the MTB peptide E7 is an ideal CD4^+^ T cell-response antigen which was detected with IFNγ-ELISPOT test and was authorized for China patent CN 101446585 A^13^. E7 comes from the early secretory antigenic target 6 (ESAT6), a type of secreted protein purified and separated from the early culture medium of MTB. Peptide C5 is obtained from the 10-kDa culture filtrate protein (CFP-10) of MTB which is known as ESAT-6-like protein encoded by the esxB gene. We extracted an MTB peptide/HLA-DR monomer from the stable cell lines established that were already expressing soluble biotinylated MTB peptide/HLA-DR monomer. E7, C5, or non-peptide tetramers constructed with different HLA-DRB1 alleles (Table 1) were used to analyze the peptide-bound CD4^+^ T cells in pleural fluid (PLF) from TB patients by MACS.

**Table 1:** Components of MTB peptide/non-peptide HLA-DR tetramers constructed with different HLA-DRB1 alleles.

Tetramer No.	Peptide-HLA-DRA^A^	HLA-DRB1 alleles^B^
1	E7	DRB1*08032
2	E7	DRB1*0818
3	E7	DRB1*0404
4	E7	DEB1*160201
9	E7	DRB1*090102
10	E7	DRB1*130201
11	E7	DRB1*150101
12	E7	DRB1*1503
6	C5	DRB1*0818
15	C5	DRB1*150101
16	C5	DRB1*1503
AK2	No peptide	DRB1*0818
AK11	No peptide	DRB1*150101
AK12	No peptide	DRB1*1503

^A^Peptide fused with HLA-DRA. ^B^HLA-DRB1 alleles of tetramers.

In addition, we observed a phenomenon of drift binding between HLA-DR alleles and TCR in our previous examinations. We used E7/HLA-DRB1*08032 and E7/HLA-DRB1*0818 tetramers to combine and detect the tetramer-bound CD4^+^ T cells in TB patients. Our findings revealed a positive rate that was not as high as expected in TB patients with a HLA-DRB1*08 allele, while it had a clearly available detection in TB patients with non-HLA-DRB1*08 allele^14^. Besides, there have been many reports of the binding drift phenomenon between HLA-DR allele and TCR^15,16^. For instance, D’Orsogna discovered the use of HLA-B*0801/EB virus tetramer to sort CD8^+^ T cells specifically and those cells could combine with artificial APC with HLA-B*4402 and HLA-B*5501 allele^17^. Therefore, in the present study, we studied the spectratyping characteristics of the MTB peptide tetramers-bound CD4^+^ TCRα- and β-chains CDR3 region in TB patients and intended to explore the possible causes and mechanisms of drift binding and antigen presentation between HLA-DRB1 alleles and CD4^+^ TCR against MTB.

## Results

### CDR3 amino-acid sequences of E7-bound CD4^+^ T cells were completely or partially identical in one of the patients

We obtained E7-bound CD4^+^ TCRα CDR3 amino-acid sequences from four TB patients (PLFs 1, 2, 5, and 12) and TCRβ CDR3 sequences from nine TB patients (PLFs 2, 3, 4, 5, 6, 7, 8, 11, and 12). The sequences contained two termini with conserved sequences at the N and C termini, i.e., YLC and FG, respectively, and the inner region exhibited diverse sequences. The comparison of nucleotide and amino-acid sequences showed that E7-bound CD4^+^ T cells with the same function shared the identical TCR α- or β-chain CDR3 sequence in one TB patient (Tables 2 and 3). In different patients, it was diverse or just shared a similar amino-acid motif. Irrespective of the patient examined, the CDR3 amino-acid sequences of both E7 and non-peptide tetramers unbound CD4^+^ T cells were quite different (Table S1). These results suggested that E7-bound CD4^+^ T cells with different HLA-DRB1 alleles might display clonal expansion in one single individual.

**Table 2:** Nucleotide and amino-acid sequences of TCRα CDR3 of E7/HLA-DR-bound CD4^+^ T cells with different HLA-DRB1 alleles.

PLF No.	Tetramer^A^	Clones	Vα^B^	CDR3 Region^C^	Jα^D^	AAN^E^
1	1/2/4/9/10	20	TACATCTGT	GCTGTGAGAGATGAAAACTATCAGTTAATCTGGGGC	GCTGGG	12
			YIC	AVRDQNYQLIWG	AG	
2	2/3/4/9/10/11/12	28	TACCTCTGT	GCAAGCAGTGGTATCAACGCAGAGTACTGGATATTGCACCATTCC	TTGGGG	15
			YLC	ASSGINAEYSILHHS	LG	
5	1/2/3/4/9/11/12	28	TACCTCTGC	GCAATGAGCGCGGGGAACCAGGGAGGCAAGCTTATC	TTTGGA	12
			YLC	AMSAGNQGGKLI	FG	
12	2	5	TACCTCTGT	GCCAGTGCGGACAGCTTTTCCTACGAGCAGTAC	TTCGGG	11
			YLC	ASADSFSYEQY	FG	

^A^Tetramers 1/2/3/4/9/10/11/12 were E7/HLA-DR tetramers with different HLA-DR alleles. ^B^Nucleotide and amino-acid sequences of V region terminal. ^C^Nucleotide and amino-acid sequences of CDR3 region. ^D^Joining nucleotide and amino-acid sequences. ^E^CDR3 amino-acid sequences’ length.

**Table 3:** Nucleotide and amino-acid sequences of TCRβ CDR3 of E7/HLA-DR-bound CD4^+^ T cells with different HLA-DRB1 alleles.

PLF No.	Tetramer^A^	Clones	Vβ^B^	CDR3 Region^C^	Jβ^D^	AAN^E^
2	1/2/3/4/9/10/11/12	40	TACTTCTGT	GCCAGCAGTGGGGACAGGAACTCTTACGAGCAGTAC	TTCGGG	12
			YFC	ASSGDRNSYEQY	FG	
3	1/2/3/4/9/10/11/12	40	TACCTCTGT	GCCAGCAGGGGGGGGGGGCCAGAAGAGGACCAGTAC	TTCGGG	12
			YLC	ASRGGGPEEDQY	FG	
4	1/2/3/4/9/10/11/12	40	TACCTATGT	GCTGCCCAGTTCCACCTGCTGCACTCCAAG	TTCCAC	10
			YLC	AAQFHLLHSK	FH	
5	1/2/3/10	20	TATCTCTGC	AGCGTTGAAGGCCAGAGGAGGGTCCCCTCTTTC	TTTGGA	11
			YLC	SVEGQRRVPSF	FG	
6	2/3/11/12	20	TATTTCTGT	GCCAGCAGCTCGTCCAGCTTGAACACTGAAGCTTTC	TTTGGA	12
			YFC	ASSSSSLNTEAF	FG	
7	1/9	10	TATCTCTGC	AGCGTTGAAGGGCAGGGGGCTAGTGGCTACACC	TTCGGT	11
			YLC	SVEGQGASGYT	FG	
8	10/12	10	TATCTCTGC	AGGGTTGAAGATGGGACAGGGGGCCAGAGTGGCTACACC	TTCGGT	13
			YLC	SVEDGTGGQSGYT	FG	
11	2/11/12	15	TACTTCTGT	GCCAGCAGTGGGGACAGGAACTCCTACGAGCAGTAC	TTCGGG	12
			YFC	ASSGDRNSYEQY	FG	
12	2	6	TACCTCTGT	GCTGTGAATGAGGACACGGGCAGGAGAGCACTTACT	TTTGGG	12
			YLC	AVNEDTGRRALT	FG	

^A^Tetramers 1/2/3/4/9/10/11/12 were E7/HLA-DR tetramers with different HLA-DR alleles. ^B^Nucleotide and amino-acid sequences of V region terminal. ^C^Nucleotide and amino-acid sequencesof CDR3 region. ^D^Joining nucleotide and amino-acid sequences. ^E^CDR3 amino-acid sequences’ length.

### The clonal expansion of E7-bound CD4^+^ T cells was due to peptide specificity rather than HLA-DR specificity

We continually studied the reason for the phenomenon that E7-bound CD4^+^ T cells with different HLA-DRB1 alleles exhibited clonal expansion in one patient. In PLF11 (Table 4), E7-bound CD4^+^ T cells with different HLA-DRB1 alleles shared an identical CDR3 amino-acid sequence (ASSGDRNSYEQY), while C5-bound CD4^+^ T cells with different HLA-DR alleles shared another one (SGLAGGAYEQY). The terminal sequences “AS” and “SA” also included in conserved sequences which translated by different primer pairs designed before. In contrast, CDR3 sequences of non-peptide bound CD4^+^ T cells were different from each other. In addition, the sequences of E7 or non-peptide/HLA-DR tetramers unbound CD4^+^ T cells were not the same each other at all (Table S1). Thus, the clonal expansion of peptide-bound CD4^+^ T cells was mostly due to peptide specificity rather than HLA-DR specificity.

**Table 4:** Nucleotide and amino-acid sequences of TCRβ CDR3 of E7/C5/non-peptide bound CD4^+^ T cells with different HLA-DRB1 alleles in PLF11.

PLF No.	Tetramer^A^	Peptide^B^	HLA-DRB allele^C^	Clones	Vβ^D^	CDR3 Region^E^	Jβ^F^	AAN^G^
11	2	E7	DRB1*0818	2	TACTTCTGT	GCCAGCAGTGGGGACAGGAACTCCTACGAGCAGTAC	TTCGGG	12
					YFC	ASSGDRNSYEQY	FG	
11	11	E7	DRB1*150101	4	TACTTCTGT	GCCAGCAGTGGGGACAGGAACTCCTACGAGCAGTAC	TTCGGG	12
					YFC	ASSGDRNSYEQY	FG	
11	12	E7	DRB1*1503	4	TACTTCTGT	GCCAGCAGTGGGGACAGGAACTCCTACGAGCAGTAC	TTCGGG	12
					YFC	ASSGDRNSYEQY	FG	
11	6	C5	DRB1*0818	5	TACTTCTGT	GCCAGCAGCGGACTAGCGGGAGGTGCCTACGAGCAGTAC	TTCGGG	13
					YFC	ASSGLAGGAYEQY	FG	
11	15	C5	DRB1*150101	2	TACATCTGC	AGTGCTAGTGGACTAGCGGGAGGTGCCTACGAGCAGTAC	TTCGGG	13
					YIC	SASGLAGGAYEQY	FG	
11	16	C5	DRB1*1503	4	TACCTCTGT	AGTGCTAGTGGACTAGCGGGAGGTGCCTACGAGCAGTAC	TTCGGG	13
					YLC	SASGLAGGAYEQY	FG	
11	16	C5	DRB1*1503	1	TATTTCTGT	GCCAGCAGTGGGGACAGGAACTCCTACGAGCAGTAC	TTCGGG	12
					YFC	ASSGDRNSYEQY	FG	
11	AK2		DRB1*0818	2	TATTTCTGT	GCCAGCAGTGAGGGGGGGGGGGACAATGAGCAGTTC	TTCGGG	12
					YFC	ASSEGGGDNEQF	FG	
11	AK2		DRB1*0818	1	TATCTCTGC	AGCGTTAGGTCCGGGACGGGACAAAATGAGCAGTAC	TTCGGG	12
					YLC	AVRSGTGQNEQY	FG	
11	AK11		DRB1*150101	2	TATTTCTGT	GCCAGCAGTGGGGACAGGAACTCCTACGAGCAGTAC	TTCGGG	12
					YFC	ASSGDRNSYEQY	FG	
11	AK11		DRB1*150101	2	TATCTCTGC	AGCGTTAGGTCCGGGACGGGACAAAATGAGCAGTAC	TTCGGG	12
					YLC	AVRSGTGQNEQY	FG	
11	AK12		DRB1*1503	3	TATCTCTGC	GCCAGCAGCTTACTGAGCTCCACCGGGGAGCTGTTT	TTTGGA	12
					YLC	ASSLLSSTGELF	FG	
11	AK12		DRB1*1503	1	TATTTCTGT	GCCAGCAGTGGGGACAGGAACTCCTACGAGCAGTAC	TTCGGG	12
					YFC	ASSGDRNSYEQY	FG	
11	AK12		DRB1*1503	1	TATCTCTGC	AGCGTTAGGTCCGGGACGGGACAAAATGAGCAGTAC	TTCGGG	12
					YLC	AVRSGTGQNEQY	FG	

^A^Tetramers 2/11/12 were E7/HLA-DR tetramers. Tetramers 6/15/16 were C5/HLA-DR tetramers. Tetramers AK2/AK11/AK12 were non-peptide HLA-DR tetramers. ^B^Peptide fused with HLA-DRA. ^C^HLA-DRB allele of tetramers. ^D^Nucleotide and amino-acid sequences of V region terminal. ^E^Nucleotide and amino-acid sequences of CDR3 region. ^F^Joining nucleotide and amino-acid sequences. ^G^CDR3 amino-acid sequences’ length.

### TCR Vα and TCR Vβ of E7-bound CD4^+^ T cells in TB patients perform dominant distribution

We chose three TB patients (PLFs 5, 6, and 11) whose E7-bound CD4^+^ T cells with different HLA-DRB1 alleles were clonal expansion and amplified their full-length nucleotide sequences of TCR α- and β-chains. BLAST software was used to analyze their V, J, and D family. The dominant TCR Vα and TCR Vβ of E7-bound CD4^+^ T cells in these patients were diverse from each other (Table 5). PLF5 was mostly on TRAV12-3 and TRBV12-3. In contrast, PLF6 was mainly on TRBV14, and PLF11 was entirely on TRBV10-1. Besides, TCR J and D family in these patients were more consistent. To sum up, different TB patients exhibited various TCR Vα and TCR Vβ profiles. Even within the same patient, the profile of each E7-bound CD4^+^ T cell with different HLA-DRB1 alleles was significantly different. Some TCR Vα and TCR Vβ gene families from TB patient showed oligoclonal.

**Table 5:** The TCR Vα and Vβ of E7-bound CD4^+^ T cells in TB patients performed dominant distribution.

PLF No.	Tetramer^A^	Clones	Vα/Vβ^B^	CDR3 Region^C^	Jα/Jβ^D^	V Family^E^	J Family^F^	D Family^G^
5	1/2/3/4/9/11/12	28	YLC	GCAATGAGCGCGGGGAACCAGGGAGGAAAGCTTATC	FG	TRAV12-3	TRAJ23	
				AMSAGNQGGKLI				
5	10	4	YLC	GCAATGAGCGCGGGGAACCAGGGAGGAAAGCTTATC	FG	TRAV12-1	TRAJ20	
				AMSAGNQGGKLI				
5	1/2/10	15	YLC	AGCGTTGAAGGCCAGAGGAGGGTCCCCTCTTTC	FG	TRBV12-3	TRBJ2	TRBD1
				SVEGQRRVPSF				
5	3	5	YLC	AGCGTTGAAGGCCAGAGGAGGGTCCCCTCTTTC	FG	TRBV18-1	TRBJ2	TRBD1
				SVEGQRRVPSF				
6	2/11	10	YFC	GCCAGCAGCTCGTCCAGCTTGAACACTGAAGCTTTC	FG	TRBV14	TRBJ2	TRBD2
				ASSSSSLNTEAF				
6	12	5	YFC	GCCAGCAGCTCGTCCAGCTTGAACACTGAAGCTTTC	FG	TRBV14	TRBJ2	TRBD1
				ASSSSSLNTEAF				
6	3	5	YFC	GCCAGCAGCTCGTCCAGCTTGAACACTGAAGCTTTC	FG	TRBV29-1	TRBJ2	TRBD2
				ASSSSSLNTEAF				
11	2/11/12	15	YFC	GCCAGCAGTGGGGACAGGAACTCCTACGAGCAGTAC	FG	TRBV10-1	TRBJ2	TRBD1
				ASSGDRNSYEQY				

^A^Tetramers 1/2/3/4/9/10/11/12 were E7/HLA-DR tetramers with different HLA-DR alleles. ^B^Amino-acid sequences of V region terminal. ^C^Nucleotide and amino-acid sequences of CDR3 region. ^D^Joining nucleotide and amino-acid sequences. ^E^Vα gene family or Vβ gene family. ^F^Jα gene family or Jβ gene family. ^G^Dβ gene family.

### The structures of TCRβ CDR3 region of E7-bound CD4^+^ T cells with different HLA-DRB1 alleles were similar

**Figure 1:**
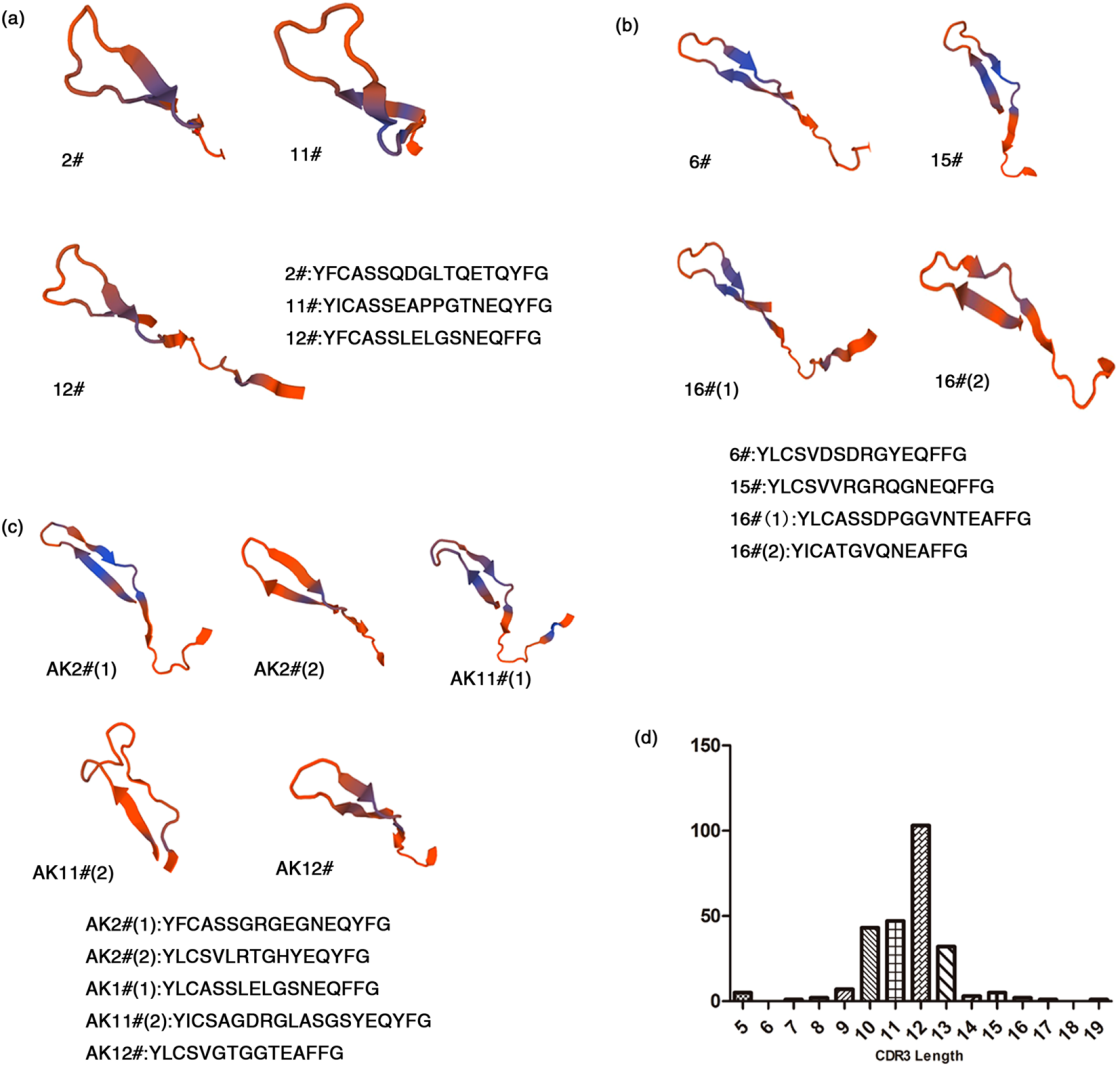
The structures of TCRβ CDR3 region of E7, C5 or non-peptide/HLA-DR tetramers-bound CD4^+^ T cells in PLF13 and comparison of amino-acid sequences’ length of TCRβ CDR3 of E7/HLA-DR-bound CD4^+^ T cells. (**a**) The protein tertiary structures of E7/HLA-DR tetramers 2#, 11#, and 12# bound CD4^+^ TCRβ CDR3 region, area between the arrows. Although amino-acid sequences are different, the CDR3 protein tertiary structure showed similar each other in PLF13 as long as the peptide E7 binds; (**b**) The protein tertiary structures of C5/HLA-DR tetramers 6#, 15#, and 16# bound CD4^+^ TCRβ CDR3 region. The structure of C5-bound CD4^+^ TCRβ CDR3 region showed another shape of structure, which was distinct from those of E7-bound CD4^+^ T cells; (**c**) The protein tertiary structures of E7/HLA-DR tetramers AK2#, AK11#, and AK12# bound CD4^+^ TCRβ CDR3 region. Non-peptide tetramers bound CD4^+^ T cells TCRβ CDR3 tertiary structures were entirely different each other. In a, b, and c, all structures were oriented with CDR3 region in the top left corner. According to the number of tetramers, structures, from left to right, corresponded to their respective amino-acid sequences; (**d**) Eleven TB patients were included in this study (clones = 252). Frequency histograms of TCRβ CDR3 amino-acid sequences were shown. For patients, the count of TCRβ CDR3 amino-acid sequences’ length of E7-bound CD4^+^ T cells in total sequence results was calculated (Y axis), which focused on 5 to 19 amino-acid residues (X axis), mainly distributed in 12 amino-acid sequences.

Since we had also observed completely different CDR3 sequences of E7-bound CD4^+^ T cells in some single one TB patient, so considered to analyze their protein tertiary structures. Taken PLF13 as a typical example, input raw amino-acid sequences containing CDR3 region into Swiss-Model Workspace (https://www.swissmodel.expasy.org/interactive) to predict protein tertiary structure, area between the arrows in the upper left was CDR3 region protein tertiary structure (Fig. 1a–c). Framework sequences were conserved, N-terminal (REFV or REFI) and C-terminal (PGTRLTVTEDLKNVF) amino-acid residues of CDR3 loop were composed of almost the same amino-acid residues. On the premise of E7 combining, in spite of the different amino-acid sequences, the CDR3 protein tertiary structure showed similar each other in PLF13 (Fig. 1a). Furthermore, we also observed C5-bound CD4^+^ TCR CDR3 protein tertiary structures showed another consistent shape but was distinct from those of E7-bound CD4^+^ TCR CDR3 region (Fig. 1b). In contrast, non-peptide tetramers bound CD4^+^ TCR CDR3 tertiary structures were entirely different each other (Fig. 1c). These results suggested that in some TB patients E7/HLA-DR tetramers with different HLA-DRB1 alleles were capable of recognizing and binding CDR3 fragments with different sequences but a similar structure and function.

### The most common length of TCRβ CDR3 of E7-bound CD4^+^ T cells is 12 amino-acid sequences

There were a few CDR3 sequences of TCR α-chain in CD4^+^ T cells so that it was not enough to statistic. Therefore, we counted the length of TCRβ CDR3 amino-acid sequences of E7/HLA-DR-bound CD4^+^ T cells with different HLA-DRB1 alleles in eleven TB patients (clones, n = 252). The counting result showed that the maximal length of CDR3 sequence consisted of 12 amino-acids, accounting for 40.873% (103 of 252 clones) (Fig. 1d). E7-bound CD4^+^ T cells displayed TCRβ CDR3 amino-acid sequences’ length dominant distribution in immune reaction against specific MTB peptide E7.

### Molecular chaperones CD3 and CD4 play a supporting role in the binding force between E7/HLA-DR tetramers and CD4^+^ T cells

CD3 and CD4 are responsible for conducting the TCR activation signal in the process of T-cell recognition of antigens. We used E7/HLA-DR tetramers to sort CD4^+^ T cells from two TB patients (PLFs 9 and 10), followed by treatment with CD3 and CD4 goat polyclonal antibodies to block them before sorting. The results showed that E7-bound CD4^+^ T cells blocked with CD3 and CD4 polyclonal antibodies exhibited disorganized CDR3 amino-acid sequences (Table 6), also different with unbound CD4^+^ T cells (Table S2). It suggested that the suppression of function of CD3 and CD4 apparently affected the process of T cell recognizing antigen.

**Table 6:** Nucleotide and amino-acid sequences of TCRβ CDR3 of CD4^+^ T cells blocked by CD3 and CD4 polyclonal antibodies in PLFs 9 and 10.

PLF No.	tetramer^A^	Clones	Vβ^B^	CDR3 Region^C^	Jβ^D^	AAN^E^
9	1B	1	TACTTCTGT	GCCAGCAGTCCTGGGACAGGGGGCCACGAGCAGTAC	TTCGGG	12
			YFC	ASSPRTGGHEQY	FG	
9	1B	2	TATCTCTGC	AGCGTTGATGGAGGGGGGTGGTGGACTGAAGCTTTC	TTTGGA	12
			YLC	SVDGGGWWTEAF	FG	
9	9B	2	TACTTCTGT	GCTGCCCAGTTCCTCCCCAGTCCCTCCAAG	TTCCAC	10
			YFC	AAQFLPSPSK	FH	
9	11B	1	TACTTCTGT	GCCAGCAGTATGAGTTACGAGACCCAGTAC	TTCGGG	10
			YFC	ASSMSYETQY	FG	
10	2B	1	TATCTCTGC	AGCGTTGGCGAGCCGGGACAGAACACCGGGGAGCTGTTT	TTTGGA	13
			YLC	SVGAPGQNTGELF	FG	
10	2B	2	TACTTCTGT	GCCAGCAGTTTATGGGGGGAAAGCACTGAAGCTTTC	TTTGGA	12
			YFC	ASSLRGESTEAF	FG	
10	2B	1	TACTTCTGT	GCCATCAGTGAGAGTGGCGGCGAAACGAATGAGCAGTTC	TTCGGG	13
			YFC	AISESGGETNEQF	FG	
10	2	2	TACCTCTGT	GCCAGCAGTTCCCCAGGGTGGGGAAACACCATATAT	TTTGGA	12
			YLC	ASSSPGWGNTIY	FG	
10	AK2B	1	TACCTCTGT	GCCAGCAGTGAATATACAGCCAATCAGCCCCAGCAT	TTTGGT	12
			YLC	ASSEYTANQPQH	FG	
10	AK2B	2	TACCTCTGT	GCCAGCAGCTTAGGAACAGGCGAGCAGTAC	TTCGGG	10
			YLC	ASSLGTGEQY	FG	

^A^Tetramers 1/2/9/11 were E7/HLA-DR tetramers with different HLA-DR alleles. “B” represented blocked by CD3 and CD4 polyclonal antibodies. ^B^Nucleotide and amino-acid sequences of V region terminal. ^C^Nucleotide and amino-acid sequences of CDR3 region. ^D^Joining nucleotide and amino-acid sequences. ^E^CDR3 amino-acid sequences’ length.

## Discussion

The CDR3 region of TCR varies among different T-cell populations and individuals. The rearrangement of the TCR CDR3 is random in healthy individuals without antigen stimulation, but specific T cells can be clonally activated and expanded by a foreign specific antigen, such as the MTB protective 16-kDa antigen and the MTB purified protein derivative (PPD) RT23^18^. Specific recognition of a peptide on a certain T cell can induce the clonal expansion of T cells which express a particular TCR V gene product. Analysis of the size and distribution pattern of CDR3 can be used to define the degree of clonality of T cells and has been widely exploited in the research of many diseases, such as myelodysplastic syndrome, cancer, parasitosis, and autoimmune diseases^19–23^. In our previous studies, we prepared peptide E7, C5, or non-peptide tetramers constructed with different HLA-DRB1 alleles. The non-peptide DRB1 tetramers in fact contain no peptides. In the case of HLA-DR typing of the defined was detected not match with the genes of tetramers, there was a certain different level of detection^14^, which revealed that the phenomenon of binding drift between HLA-DR and CD4^+^ TCR could lead to elucidating the possible causes and mechanisms of CD4^+^ T cells related presentation against MTB.

The CDR3 regions of TCR α- and β-chains are responsible for recognition of the antigenic peptides presented to the CD4^+^ T cell by HLA class II. As antigen-MHC complex, MTB peptide/HLA-DRB1 tetramers bind to CDR3 region of CD4^+^ TCR. MTB peptide directly combined with the middle region of CDR3, while the conserved flanking regions bind to the side walls of antigen-binding-groove of MHC molecules. Consequently, there needs to be a high level of diversity in CDR3 region, which is generated by varying both the sequences and the number of amino-acids. In this study, we found lengths of E7-bound CD4^+^ TCRβ CDR3 amino-acid sequences mainly on 12. Some publications also reported the characteristics of the CDR3 lengths’ distribution of T cells or B cells in diseases, such as cancer, myelodysplastic syndrome, infectious, and autoimmune disorders^24–27^. Additionally, several reports indicated that the CDR3 sequence of antigen-specific T cells recognizing the same peptide shared identical CDR3 amino-acid motifs^28–30^, but no evidence has been reported that the CDR3 sequences of the same MTB peptide-bound CD4^+^ T cells sorted with the peptide tetramers with different HLA-DRB1 alleles are the same. In this study, we found that E7-bound CD4^+^ T cells with different HLA-DRB1 alleles shared one specific CDR3 sequence of TCR α- or β-chains in a single individual. We speculated that the E7/HLA-DR tetramers with different HLA-DR alleles could be identified by the CD4^+^ T cells that had the same function and an identical sequence of the CDR3 region in one TB patient. Nevertheless, that the TCR CDR3 amino-acid sequence was diverse in different patients suggested that E7/HLA-DR tetramers with different HLA-DR alleles might be identified by those CD4^+^ T cells with the same function but the different CDR3 sequences in different patients. Overall, the same MTB peptide bound CD4^+^ T cells sorted with the peptide tetramers with different HLA-DRB1 alleles showed clonal expansion that performed the identical CDR3 sequence and amino-acid sequences’ length dominant distribution in TB patients.

Then we found that the clonal expansion of CD4^+^ T cells was due to peptide specificity rather than HLA-DR specificity. However, in a few cases, the clonal expansion of CD4^+^ T cells to a certain extent is dependent on HLA-DR restriction. Moreover, we genotyped the HLA-DR type of PLF11 in order to further analyze the effect of patient’s HLA-DR allele background on CD4^+^ T cells in combination with peptide/HLA-DR tetramers constructed with different HLA-DR alleles. The MTB peptide/HLA-DR tetramers with different HLA-DRB1 alleles (DRB1*0818, DRB1*150101, and DRB1*1503) not completely matching PLF11’s genetic background with HLA-DRB1*0101 and HLA-DRB1*0803 were capable of binding CD4^+^ T cells with the identical CDR3 amino-acid sequence in PLF11. Although the E7/HLA-DRB1*0818 and C5/HLA-DRB1*0818 tetramers shared the same classification of HLA-DRB1*08 allele with PLF11, the CDR3 sequence of E7 or C5/HLA-DRB1*08 tetramers bound CD4^+^ T cells did not show any specific results. Likewise, E7/HLA-DRB1*0818 tetramer bound CD4^+^ T cells in PLF12 with HLA-DRB1*1101 and HLA-DRB1*1302 displayed the consistent CDR3 sequence only at a suitable concentration of the tetramer. All these results provided the novel discovery of antigen presented process between CD4^+^ T cells and MHC class II in reference to HLA-DR type. Besides, CD8^+^ T cells also play a part in the protective immunity against MTB infections, and several reports showed similar findings that the MTB antigen reactive CD8^+^ T cells doing their jobs independent of MHC restriction^31,32^. In other areas of microorganisms, Roider et al. even put forward an idea that a new CD8^+^ T cell response towards the mutated antigen which caused HIV evade phenomenon could be generated in a population not selected for certain HLA alleles^33^. However, no one has studied the detailed CDR3 sequences as we did.

Tully *et al*. demonstrated that there are individualized skewed spectratyping in the TCR of TB patients^34^. TCR repertoire can reflect the function, status, and composition of CD4^+^ T cells. In this study, we detected the spectratyping of α- and β-chain in three patients, which showed some TCR Vα and Vβ gene families of E7-bound CD4^+^ T cells were oligoclonal. Some reports evidenced that during the process of immune response to the MTB antigen, certain gene families had become predominant, which restricted the expression of other gene families^35–37^, which is consistent with our findings. Not only is the TCR Vα and Vβ gene families of E7-bound CD4^+^ T cells were oligoclonal, the CDR3 amino-acid sequences were also identical, which proved powerfully that the CD4^+^ T cells against one specific MTB antigen in one single individual were clonal expansion and their CDR3 region plays an important role in immune response against this specific antigen. Moreover, in one of the participants, we also observed the presence of several E7-bound CD4^+^ T cells with different amino-acid sequences in the CDR3 region. In addition, E7-bound CD4^+^ T cells shared one CDR3 amino-acid sequence with unbound CD4^+^ T cells in PLF12. Several possible reasons may explain these observations. First, the different CDR3 sequences likely recognize antigens with the same size and similar spatial structures. Second, the pathogenicity of MTB and the molecular weight of the antigen, as well as the types of Ig, scattered or additional antigen sites, and variability of the MTB antigen composition could contribute to the diversity established. Third, the specificity between E7 and CD4^+^ T cells might primarily depend on the flanking sequence of CDR3, which might be depend on a small number of key amino-acids. Fourth, patient-specific factors may also contribute to the degree of CDR3 variation against a specific MTB antigen.

The TCR antigen-binding loops use different conformations in their interaction with each distinct ligand^38^. In our examination, not every TB patients showed the identical CDR3 sequence of E7-bound CD4^+^ T cells. Importantly, we also found completely different CDR3 sequences in some of the TB patients, whose architectural properties were consistent or not aroused our interest. In PLF13, the protein tertiary structure of the CD4^+^ TCR β-chain CDR3 region showed similar each other, in spite of the completely different amino-acid sequences. These structures provided unique insights into the mechanisms of MTB antigen recognition between CD4^+^ T cells and MHC. E7/HLA-DR tetramers with different HLA-DRB1 alleles were capable of recognizing and binding CDR3 fragments with an identical sequence or with different sequences but a similar structure and function in one individual. In different TB patients, the diverse CDR3 sequences of E7-bound CD4^+^ T cells seemed to show mostly similar protein tertiary structures (Fig. S1a). By contrast, the diverse CDR3 sequences of non-peptide tetramers bound CD4^+^ T cells displayed almost different protein tertiary structures (Fig. S1b–d). We suggested that CDR3 region combined with an identical peptide, such as E7 or C5, had the same function and a similar complementary groove even if found in different TB patients. We imagine that each non-peptide/HLA-DRB1 tetramers combined with CD4^+^ T cells in a nonspecific binding manner. When the conserved flanking regions bind to the side walls of antigen-binding-groove of MHC molecules and no peptide combine with TCRαβ CDR3 region, the rearrangement of the TCR CDR3 is random and the sequences are messy. Thus, after the release of its specific peptide, the CDR3 region became oddly shaped. Furthermore, we compared the amino-acid properties of E7/C5/non-peptide bound CD4^+^ T cells β-chain CDR3 region in PLF13 and found no difference between the three types of CD4^+^ T cells, regardless of whether it was hydrophilic or hydrophobic; acidic, alkaline, or neutral. When we blocked the molecule CD3 and CD4 on the surface of CD4^+^ T cells, the amino-acid sequences of the CDR3 region in E7-bound CD4^+^ T cells were scattered. Thus, we believed that molecular chaperones CD3 and CD4 could assist in TCR recognition of the antigen. Therefore, to minimize the impact on the specific binding between antigen-MHC complex and TCRs by molecular chaperones due to the effect of CD3 and CD4, it is important that the dosage of CD3 and CD4 antibodies need to be carefully determined when used in combination with peptide/MHC tetramer, and special care should be taken in the experimental operation.

In conclusion, our findings reveal that in one individual, the MTB peptide E7/HLA-DR tetramers constructed with different HLA-DRB1 alleles can bind to CD4^+^ T cells due to sharing the same CDR3 amino-acid sequence or a similar CDR3 structure and function, which suggests that E7-bound CD4^+^ T cells with different HLA-DRB1 alleles undergo clonal expansion in TB patients. These results indicate that the CDR3 region of CD4^+^ T cells is a key region for the recognition of a specific MTB antigen. The predominant TCR α- and β-chain CDR3 sequence exhibit peptide specificity, and there is also certain HLA-DR restriction which may provide a clue of the possible causes and mechanisms of peptide-specific CD4^+^ T cell-related presentation against MTB in different TB patients with different HLA-DR alleles.

## Materials and Methods

### Ethics and methods statements

The recruitment process, delivery and use of clinical samples obtained from TB patients, and the experimental procedures were approved by the Medical Ethics Committee of Zhongshan School of Medicine, Sun Yat-sen University, Guangzhou, China (Protocol number 2012-28). All of the patients provided written informed consent before enrollment in the study. And all methods and experiments were performed in accordance with the relevant guidelines and regulations.

### Patients and collection of samples

In the present investigation, patients diagnosed with tuberculosis pleurisy were recruited from the Guangzhou Chest Hospital, Guangzhou City, Guangdong Province, China. The diagnosis of active tuberculosis pleurisy was made based on the following criteria: (1) positive pleural fluid smear or culture results for MTB; (2) detection of active tuberculosis pleurisy lesions by X-ray examination or pleural biopsy sections by MTB antigen-specific immunohistochemistry; and (3) the presence of typical systemic and local symptoms, such as fever, chills, sweating, loss of appetite, fatigue, night sweats, chest distress, chest pain, cough, and short breath or dyspnea. The possibility of a malignant lesion of the lung or lymph node was ruled out. PLF and peripheral blood samples were collected from inpatients that were untreated or in the initial stage of treatment.

### Preparation of MTB peptide or non-peptide/HLA-DR tetramers

Stable drosophila S2 cell lines expressing soluble biotinylated MTB peptide or non-peptide/HLA-DR monomers were established, and then biotinylated monomers were expressed and purified for building tetramers as described earlier^14,39^. MTB peptide or non-peptide/HLA-DR tetramers were obtained by mixing the corresponding monomers with PE-labeled streptavidin (eBioscience) in an 8:1 molar ratio at room temperature for MACS.

### Obtaining MTB peptide or non-peptide/HLA-DR tetramers bound CD4^+^ T cells

Plural fluid cells samples were isolated by Ficoll-Hypaque (Axis-Shield) density gradient centrifugation and were suspended to a final concentration of 1 × 10^6^ cells/mL in complete RPMI 1640 medium (Gibco). CD4^+^ T cells were purified from 1 × 10^7^ of PFCs in 40 μL sorting buffer using 10 μL of CD4^+^ T-cell Negative Sorting Microbeads (Miltenyi Biotec). When required, 2 μL CD3 and 2 μL CD4 goat polyclonal antibodies (Santa Cruz Biotechnology) were used to block the action site before sorting by E7/HLA-DR tetramers in total 50 μL sorting solution. Then, MTB peptide or non-peptide/HLA-DR tetramers bound CD4^+^ T cells were purified from CD4^+^ T cells using final concentration 6 μg/mL of MTB peptide or non-peptide/HLA-DR tetramers labeled with PE prepared before and Anti-PE MicroBeads (Miltenyi Biotec, 10 μL added in 90 μL sorting solution). Meanwhile, the cells that were not in combination with Anti-PE magnetic beads namely unbound CD4^+^ T cells were also preserved.

### Extraction of RNAs and synthesis of the first cDNAs

Using TRIzol reagent (Invitrogen), total RNA was extracted from CD4^+^ T cells obtained before the experiment. Total RNA was reverse-transcribed into cDNA by a reverse transcription-polymerase chain reaction (RT-PCR) using SuperScript TM III Reverse Transcriptase kit (Invitrogen). Double-stranded cDNA was synthesized by long-distance polymerase chain reaction (LD-PCR) with the primers and reagents provided from the BD SMART PCR cDNA Synthesis Kit (Agilent). The LD-PCR products were stored in −20 °C and utilized as DNA templates for cloning and sequencing of the CDR3 region and V region of TCR α- and β-chain by multiple PCR reactions.

### Cloning and sequencing of TCR α- and β-chain CDR3 region and V family

TCR CDR3 size analysis of each sample was performed by multiple PCR amplification. We used mixed primer pairs to perform multiple PCR amplification. Each group of mixed primer pairs contains 1 to 5 forward primers and one reverse primer. One clone represents the CDR3 sequence successfully amplified by using one group of mixed primer pair. The reactions for each of the samples were carried out in 20 μL mixtures that contained 0.4 μL of a sense primer and 0.4 μL of an antisense primer, 10 μL of a Taq polymerase mix (Genestar), 1 μL of DNA template, and 8.2 μL of ddH_2_O. The experimental conditions consisted of the following cycles: 95 °C for 5 min, melting at 95 °C for 30 s, primer annealing at 60 °C for 30 s, 72 °C for 1 min (30 cycles), followed by extension at 72 °C for 1 min. Additionally, TCR V size analysis of each sample was performed by PCR amplification. The reaction for each samples were performed in 25 μL mixtures that included 1 μL of the sense primer and 1 μL of the antisense primer, 0.125 μL of Go-Taq flexi polymerase (Promega), 3 μL of Mg^2+^ (Promega), 0.5 μL of dNTP (Promega), 1 μL of a DNA template, 5 μL 5 × buffer and 12.375 μL of ddH_2_O. The experimental program contained the following cycles: 95 °C for 5 min, melting at 95 °C for 50 s, primer annealing at 63 °C for 1 min, 72 °C for 1 min (30 cycles), followed by extension at 72 °C for 5 min. Finally, all of the PCR products were separated on a 1% agarose gel (Biowest), stained with SYBR dye (Probe Bioscience), and purified using gel extraction kits (Omega). The purified DNA fragments were ligated into the pGEM-T easy vector (Promega), and the resulting plasmids were transfected by heat shock into competent *Escherichia coli* DH5h (TransGen Biotech) for propagation. The colonies which were positive for ampicillin resistance were suspended and analyzed by the Beijing Genomics Institute (Beijing, China).

### Genotyping of HLA-DR alleles of selected typical TB patients

Peripheral blood samples were collected from two typical diagnosed TB patients (PLFs 11 and 12), and genotyping of HLA-DR alleles was performed, by the Beijing Genomics Institute. The HLA sequence-based typing (SBT) approach was adopted to obtain full-length coding sequences, and the results were compared in the IMGT/HLA database to define alleles.

### Statistics

Data were statistically analyzed by using Lasergene and DNAstar software. We compared the nucleotide sequences in the IMGT/TCR database on the International ImMunoGeneTics information system website ® (IMGT/V-QUEST, http://www.imgt.org/IMGT_vquest/share/textes/). Then, we analyzed their V-(D)-J subgroup type and CDR3 region spectral type of TCR α- and β-chains, respectively. Raw CDR3 amino-acid sequences were put into the Swiss-Model Workspace (https://www.swissmodel.expasy.org/interactive) to predict tertiary protein structure.

## Electronic supplementary material

Supplementary information
