# Supplementary material for: Mycobacterium tuberculosis peptide E7/HLA-DRB1 tetramers with different HLA-DR alleles bound CD4+ T cells might share identical CDR3 region
Source: Sci Rep. 2018 Jul 2;8:9903. doi: 10.1038/s41598-018-28344-7 (PMC6028479; doi:10.1038/s41598-018-28344-7)
Supplement: Supplementary file 1 — Supplementary information [file 41598_2018_28344_MOESM1_ESM.pdf]

Mycobacterium tuberculosis peptide E7/HLA-DRB1 tetramers with different HLA-DR alleles bound CD4$^+$ T cells might share identical CDR3 region

Yichuan Gan[1,2,3,#], Cong Wang[1,2,3,#], Yimin Fang[4,#], Yanan Yao[1,2,3], Xiaoxin Tu[1,2,3], Jiao Wang[1,2,3], Xi Huang[2,5], Yaoju Tan[4], Tao Chen[6], Kouxing Zhang[7], Yanming Shen[4], Lin Zhou[6,*], Jianxiong Liu[4,*], and Xiaomin Lai[1,2,3,*]

[1]Department of Microbiology, Zhongshan School of Medicine, Sun Yat-sen University, 74 Zhongshan Road II, Guangzhou 510080, China

[2]China Ministry of Education Key Laboratory of Tropical Diseases Control; Tuberculosis Research Institute, Zhongshan School of Medicine, Sun Yat-sen University, 74 Zhongshan Road II, Guangzhou 510080, China

[3]Gangdong Provincial Department of Education Key Laboratory of Functional Molecules from Marine Microorganisms; Gangdong Provincial Research Center for Severe Infectious Disease Prevention and Control Technology, Zhongshan School of Medicine, Sun Yat-sen University, 74 Zhongshan Road II, Guangzhou 510080, China

[4]State Key Laboratory of Respiratory Disease of China, Guangzhou Chest Hospital, 62 Hengzhigang Road, Guangzhou 510095, China

[5]Department of Immunology, Zhongshan School of Medicine, Sun Yat-sen University, 74 Zhongshan Road II, Guangzhou 510080, China

[6]Tuberculosis Control Center of Guangdong Province, 485 West Huangpu Avenue,

Guangzhou 510630, China

[7]Third Affiliated Hospital of Sun Yat-sen University, 600 Tianhe Road, Guangzhou 510630, China

[#]These authors contributed equally to this work.

*Corresponding authors:

Dr. Xiaomin Lai, Department of Microbiology, Zhongshan School of Medicine, Sun Yat-sen University, 74 Zhongshan Road II, Guangzhou 510080, China, Phone/Fax: 86-20-87334126, Email: laixm@mail.sysu.edu.cn;

Dr. Jianxiong Liu, Guangzhou Chest Hospital, 62 Hengzhigang Road, Guangzhou 510095, China, Phone/Fax: 86-20-83496200, Email: Ljxer64@qq.com;

Dr. Lin Zhou, Tuberculosis Control Center of Guangdong Province, 485 West Huangpu Avenue, Guangzhou 510630, China, Phone/Fax: 86-20-38903423, Email: 13332864810@vip.163.com

Conflict-of-interest disclosure: The authors declare no conflict of interest.

Supplementary tables, and figures and figure legends

Supplementary Tables

Supplementary Table S1. Nucleotide and amino-acid sequences of TCRβ CDR3 of E7 or non-peptide tetramers unbound CD4^{+} T cells in each TB patients.

PLF No.	tetramer[A]	Vβ[B]	CDR3 Region[C]	Jβ[D]	AAN[E]
5	1’	TATCTCTGC<br>YLC	GCCAGCAGTCGGACGGACATTGACCCCCTACGAGCAGTACTTCGG<br>ASSRTDIDPLRAVLR	GCCGGG<br>AG	15
5	1’	TACCTCTGT<br>YLC	GCCAGCGATTCGGGGGTTGAAGCTTTC<br>ASDSGVEAF	TTTGGA<br>FG	9
5	2’	TATCTCTGC<br>YLC	GCCAGCAGTCAATCAAAGAGC<br>ASSQSKS	GCGGGA<br>AG	7
5	2’	TATCTCTGC<br>YLC	GCCAGCAGTCAGCCT<br>ASSQP	TCTGGG<br>SG	5
6	3’	TATCTCTGC<br>YLC	AGCGCCGGATGGGGCGGAGGGACCCAGTAC<br>SAGWGGGTQY	TTCGGG<br>FG	10
6	3’	TATCTCTGC<br>YLC	AGCGTTGAAGATATCACAGCTGATGATTTCAATGAGCAGTTC<br>SVEDITADDFNEQF	TTCGGG<br>FG	14
6	11’	TATCTCTGC<br>YLC	AGCGTGGGACGGGGGAACACTGAAGCTTTC<br>SVGRGNTEAF	TTTGGA<br>FG	10
8	1'	TATCTCTGC<br>YLC	AGCACTGAGCGGGATAGAGATACGCAGTAT<br>STERDRDTQY	TTTGGC<br>FG	10
8	9'	TATCTCTGC<br>YLC	AGCGTTGAAAACCGACTTGGCGGGGAGACCCAGTAC<br>SVENRLGGETQY	TTCGGG<br>FG	12
8	9'	TATCTCTGC<br>YLC	AGCGTTGGTTCCGGGCTAAGGGATACGCAGTAT<br>SVGSGLRDTQY	TTTGGC<br>FG	11
11	2'	TACTTCTGT<br>YFC	GCCAGCAGTGAACTTAGCGGGAGGTTATACGAGCAGTAC<br>ASSELSGRLYEQY	TTCGGG<br>FG	13
11	AK2'	TATTTCTGC<br>YFC	GCCACCTTTAATAAATCCGGGGACTGCAGCGTT<br>ATFNKSGDCSV	TTTGGA<br>FG	11
11	AK11’	TATCTCTGC<br>YLC	GCCAGCAGCCAAGCCGGGACGGGGGGGGACGAGCAGTAC<br>ASSQAGTGGDEQY	TTCGGG<br>FG	13
11	AK11’	TATCTCTGT<br>YLC	GCCAGCAGCCAAGCCGGGACGGGGGGGGACGAGCAGTAC<br>ASSQAGTGGDEQY	TTCGGG<br>FG	13
11	12’	TATATCTGC<br>YIC	GCAGTGTTTACCAAGCGGACTAATGAAAACATTTTT<br>AVFTKRTNENIF	TTTGGC<br>FG	12
11	AK12’	TATCTCTGT<br>YLC	GCCAGCAGCCGGAGCCCGGGACAAACTGAAGCTTTC<br>ASSRSPGQTEAF	TTTGGA<br>FG	12

[A]Tetramers 1/2/3/4/9/10/11/12 were E7/HLA-DR tetramers with different HLA-DR alleles. Tetramers AK2/AK11/AK12 were non-peptide HLA-DR tetramers. Symbol (’) represented unbound CD4^{+} T cells corresponding to the tetramer. [B]Nucleotide and amino acid sequences of V region terminal. [C]Nucleotide and amino acid sequences of CDR3 region. [D]Joining nucleotide and amino acid sequences. [E]CDR3 amino acid sequences’ length.

Supplementary Table S2. Nucleotide and amino-acid sequences of TCRβ CDR3 of unbound $CD4^+$ T cells blocked by CD3 and CD4 polyclonal antibodies in PLF9 and PLF10.

PLF No.	tetramer[A]	Vβ[B]	CDR3 Region[C]	Jβ[D]	AAN[E]
9	1B’	TATTTCTGT	GCCTGGAAGGGCGGACACATCTCGAACACCGGGGAGCTGTTT	TTTGGA	14
		YFC	AWKGGHISNTGELF	FG	
9	1B’	TATCTCTGC	AGCGTTGAAGTACCCAGGACAGGGTGGGCCATTCAGTAC	TTCGGC	13
		YLC	SVEVPRTGWAIQY	FG	
9	1B'	ATTCTCTGT	GCCAGCACCAGCAGTACGAGTTACGACAAGCAGTAC	TTCGGG	12
		ILC	ASTSSTSYDKQY	FG	
9	1B’	TATCTCTGC	AGCGTTGATGGAGGGGGGTGGTGGACTGAAGCTTTC	TTTGGA	12
		YLC	SVDGGGWWTEAF	FG	
9	9B’	TATTTCTGT	GCCAGTAGTGAACCGTGGGCCGGGGAGCTGTTT	TTTGGA	11
		YFC	ASSEPWAGELF	FG	
9	9B’	TATCTCTGC	AGCGTTGATGGAGGGGGGTGGTGGACTGAAGCTTTC	TTTGGA	12
		YLC	SVDGGGWWTEAF	FG	
9	11B’	TATCTCTGC	AGCGTTGATGGAGGGGGGTGGTGGACTGAAGCTTTC	TTTGGA	12
		YLC	SVDGGGWWTEAF	FG	
9	11B'	CCCGAAGTA	CTGGGTCTCGTAACTCATACTGCTGGCACA	GAGGTA	10
		YLC	ASSMSYETQY	FG	
9	11B'	TACTTCTGT	GCCAGCAGTATGAGTTACGAGACCCAGTAC	TTCGGG	10
		YFC	ASSMSYETQY	FG	
10	2B’	TATCTCTGC	AGCGTTGATCAGGGAATCCTCTACAATGAGCAGTTC	TTCGGG	12
		YLC	SVDQGILYNEQF	FG	
10	2B’	TATCTCTGC	AGCGTTGAGGGGGGGTGGGGGTACCAAGAGACCCAGTAC	TTCGGG	13
		YLC	SVEGGWGYQETQY	FG	
10	2’	TATCTCTGC	AGCGTTGAGGGGGGGTGGGGGTACCAAGAGACCCAGTAC	TTCGGG	13
		YLC	SVEGGWGYQETQY	FG	
10	2’	TATCTCTGC	AGCGTTGAAGATCGAGGTGGGTCCGCTGGCTACACC	TTCGGT	12
		YLC	SVEDRGGSAGYT	FG	
10	AK2’	TACCTCTGT	GCCAGCAGTGAATATACAGCCAATCAGCCCCAGCAT	TTTGGT	12
		YLC	ASSEYTANQPQH	FG	
10	AK2’	TACCTCTGT	GCTAGCAGGTGGGACAGGAGGGGAACAGATACGCAGTAT	TTTGGC	13
		YLC	ASRWDRRGTDTQY	FG	
10	AK2’	TACCTCTGT	GCCAGCCTCGGACAACCAACTAGAGAGCAGTAC	TTCGGG	11
		YLC	ASLGQPTREQY	FG	
10	AK2’	TACCTCTGT	GCCAGCACCGAGGAAGGGTGGGATTACGAGCAGTAC	TTCGGG	12
		YLC	ASTEEGWDYEQY	FG	
10	AK2’	TATCTCTGT	GCCACCAGTGCCCGGACACCTAACAATGAGCAGTTC	TTCGGG	12
		YLC	ATSARTPNNEQF	FG	
10	AK2’	TACCTGTGT	GCCAGCAGCTTAGGTCCCCCGGGACAGGGGGCCTACGAGCAGTAC	TTCGGG	15
		YLC	ASSLGPPGQGAYEQY	FG	

[A]Tetramers 1/2/9/11 were E7/HLA-DR tetramers with different HLA-DR alleles. Tetramers AK2/AK11/AK12 were non-peptide HLA-DR tetramers. “B” represented blocked by CD3 and CD4 polyclonal antibodies. Symbol (’) represented unbound $CD4^+$ T cells corresponding to the tetramer. [B]Nucleotide and amino acid sequences of V region terminal. [C]Nucleotide and amino acid sequences of CDR3 region. [D]Joining nucleotide and amino acid sequences. [E]CDR3 amino acid sequences’ length.

Supplementary Figures and figure legends

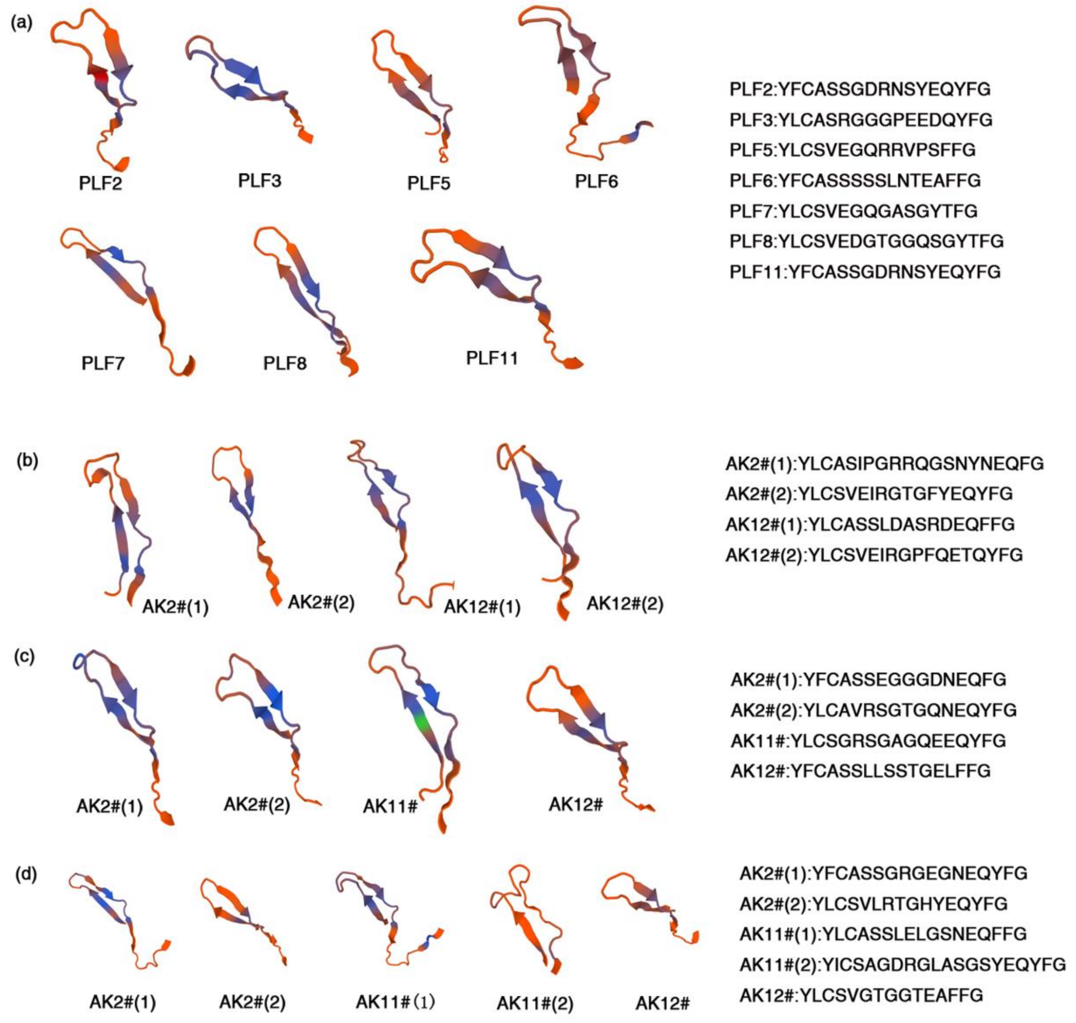

Supplementary Figure S1. Comparison of structures of E7 or non-peptide/HLA-DR tetramers-bound CD4^{+} T cells TCRβ CDR3 region in different TB patients. (a) The protein tertiary structures of E7-bound CD4^{+} TCR β chain CDR3 region, area between the arrows, in PLFs 2, 3, 5, 6, 8, 9 and 11. In different TB patients, their diverse CDR3 sequences of E7-bound CD4^{+} T cells seemed to show mostly similar protein tertiary structures; **(b)** The protein tertiary structures of AK2#, AK11# and AK12# tetramers bound CD4^{+} TCRβ CDR3 region in PLF10; **(c)** The protein tertiary structures of AK2#, AK11# and AK12# tetramers bound CD4^{+} TCRβ CDR3 region in PLF11; **(d)** The protein tertiary structures of AK2#, AK11# and AK12# tetramers bound CD4^{+} TCRβ CDR3 region in PLF13. The results of b, c, and d showed that the diverse CDR3 sequences of non-peptide tetramers bound CD4^{+} T cells displayed almost different protein tertiary structures. In **a-d**, all structures were oriented with CDR3 region in the top left corner. According to the number of PLFs or tetramers, structures, from left to right, corresponded to their respective amino-acid sequences.
